# A Comparison of Operative Time Between Robotic Surgical Assistant (ROSA) and MAKO Robotic-Assisted Total Knee Arthroplasty: A Meta-Analysis

**DOI:** 10.7759/cureus.92933

**Published:** 2025-09-22

**Authors:** Brandon Weissman, Tiger Y Ma, Kevin Shen, Shafayath Chowdhury, Douglas Speer, Constantino Lambroussis

**Affiliations:** 1 Otolaryngology, Lake Erie College of Osteopathic Medicine, Elmira, USA; 2 Neurology, Lake Erie College of Osteopathic Medicine, Greensburg, USA; 3 Medical School, Lake Erie College of Osteopathic Medicine, Greensburg, USA; 4 Anesthesiology and Critical Care, Lake Erie College of Osteopathic Medicine, Elmira, USA; 5 Internal Medicine, Lake Erie College of Osteopathic Medicine, Elmira, USA; 6 Osteopathic Manipulative Medicine, Lake Erie College of Osteopathic Medicine, Elmira, USA

**Keywords:** mako, ortho surgery, robot-assisted tka, rosa knee system, tka

## Abstract

Robotic-assisted total knee arthroplasty (TKA) represents a modern surgical approach that seeks to enhance implant precision and alignment compared to traditional methods. The Robotic Surgical Assistant (ROSA) and MAKO platforms represent two prominent systems used in robotic surgery. A systematic search of PubMed and Embase was performed through December 2024 to identify comparative studies of ROSA and MAKO robotic systems in TKA. Eligible studies reported operative time outcomes. Mean differences (MDs) with 95% confidence intervals (CIs) were calculated for each study. Pooled analyses were conducted using both fixed-effect and random-effects models. Heterogeneity was assessed using the Q statistic and I². Three studies met the inclusion criteria, with a combined sample of 1,222 patients. The fixed-effects model showed a small but statistically insignificant pooled effect favoring ROSA (Hedges’ g = 0.21, standard error = 0.07, 95% CI = -0.07 to 0.50), corresponding to approximately 6-7 minutes shorter operative time. The random-effects model demonstrated a similar trend, though non-significant due to high heterogeneity (Q = 20.07, p < 0.001, I² = 90%). Funnel plot analysis revealed no evidence of publication bias. The meta-analysis indicates that ROSA might result in shorter surgical times than MAKO during TKA procedures, although statistically insignificant. The results are restricted by substantial heterogeneity between studies and the limited number of available studies.

## Introduction and background

Total knee arthroplasty (TKA) is a common procedure in the United States, with approximately 400,000 primary procedures performed annually [1]. The surgery involves replacing damaged or diseased knee components with artificial parts. In orthopedics, TKA is one of the most successful surgeries, with most patients reporting significant pain relief, functional improvement, and an enhanced quality of life [1]. TKA is most commonly indicated in patients with end-stage osteoarthritis, a condition that is progressive and leads to degeneration of articular cartilage within the knee joint [1].

Traditional TKAs are widely regarded as successful. Still, even with this robust history of reliability, the development of robotic TKA was introduced to reduce inaccuracies in implant placement and alignment [2]. Over the past decade, robotic-assisted devices have become more prevalent in TKAs. Two popular TKA robotic systems are the Robotic Surgical Assistant (ROSA) knee system from Zimmer Biomet, Warsaw, IN, and the MAKO surgery robot from Stryker.

The ROSA, which was initially introduced in Australia in 2018, is a widely adopted robotic system for TKA [3]. The ROSA surgical robot utilizes two-dimensional (2D) X-rays to create a three-dimensional (3D) model of the patient’s knee or an image-less option based on intraoperative landmarks [4]. The ROSA platform comprises two main components: a robotic unit, which houses the robotic arm, and an optical unit, featuring an infrared camera [4]. The procedure requires the installation of two rigid bodies, in which trackers are placed, allowing for the registration of bony landmarks in 14 positions [4]. After the bony landmarks are recorded, the ROSA system assesses knee frontal laxity [4]. Once these measurements are finalized, the system uses several factors and predictive values of the final gaps and alignment, which are then given to the surgeon [3]. Finally, the ROSA robotic arm moves the jig to the determined position. Once the cut is made, the ROSA system validates the cut to confirm its accuracy [4].

The MAKO robotic arm-assisted system was launched for primary TKA in 2017 [3]. The MAKO system is composed of the robot itself, as well as an infrared tower [5]. The Mako system begins with preoperative CT planning, which enables the surgeon to have accurate 3D anatomic references [5]. During the surgery, navigational pins are placed that register the patient’s specific anatomy. The surgeon then stresses the knee in predetermined poses, which creates the gap distance [5]. A surgical plan is presented to the surgeon, and then the robotic arm enters the surgical field, making the predetermined cuts [5].

These systems are both semi-active robotic systems for TKA. They differ in that the MAKO utilizes preoperative CT images of the lower limbs [5]. Whereas the ROSA can produce a 3D image from conventional radiographs or conduct imageless procedures based on landmarks [4]. Additionally, the MAKO robotic arm utilizes haptic feedback, holds the saw blade for cutting, and stops cutting when the preoperative plan parameters are met [6]. The ROSA robotic arm has a jig attached, on which the surgeon makes the cut based on the jig’s position [4].

The variables investigated in this meta-analysis include mean operative time, the Knee Society Score (KSS), and the Hip-knee-ankle (HKA) angle. The mean operative time represents the time the TKA took. The KSS is a widely used clinical outcome measure that evaluates both the objective clinical findings (e.g., range of motion, alignment, and stability) and patient-reported functional capacities before and after TKA. It facilitates a comprehensive assessment of pain, function, and overall knee performance, helping clinicians gauge the success of TKA procedures [7]. The HKA angle is a key radiographic measure for evaluating lower-limb coronal alignment in TKA. It is typically obtained from a standing long-leg radiograph that connects the center of the femoral head, the midpoint of the knee joint, and the center of the talus, allowing clinicians to determine how effectively TKA restores the mechanical axis of the limb [8].

## Review

Methodology

Search Strategy

A systematic search of electronic databases Embase and PubMed was conducted to identify studies that included analysis of the ROSA and/or MAKO. The search utilized a combination of keywords, including “Mako and total knee arthroplasty” and “Rosa and total knee arthroplasty,” to retrieve relevant studies. Searches were limited to English-language studies published up to December 2024. Duplicate records were removed using Rayyan software (Rayyan, Cambridge, MA, USA), and the remaining studies were organized and managed using Microsoft Excel (Microsoft® Corp., Redmond, WA, USA).

Study Selection

The inclusion criteria included all study designs that used the ROSA and/or MAKO robot for TKA. Exclusion criteria were studies not published in the English language, narrative reviews, systematic reviews, guidelines, and conference proceedings. Studies not reporting relevant outcomes or that were not specified were also excluded.

Data Extraction

Two reviewers reviewed abstracts from the initial search. Once this was complete, we were left with 41 articles, from which data were extracted to a standardized spreadsheet. Extracted data included study characteristics (authors, publication year, country, study design, number of patients, and robot used). The primary outcome was the mean operative time.

Data Analysis

Data extraction and statistical analysis were performed using Microsoft Excel and supplementary statistical software. Study-level means, standard deviations, and sample sizes were used to calculate mean differences (MDs) with corresponding standard errors and 95% confidence intervals (CIs). Both fixed-effect and random-effects models were applied to generate pooled estimates, and heterogeneity was assessed using the Q statistic and I² index. One article gave the median time data for both robots; the means were obtained by using the standard deviation and reversing the log transformation to yield the means.

Summary of Included Articles

**Figure 1:**
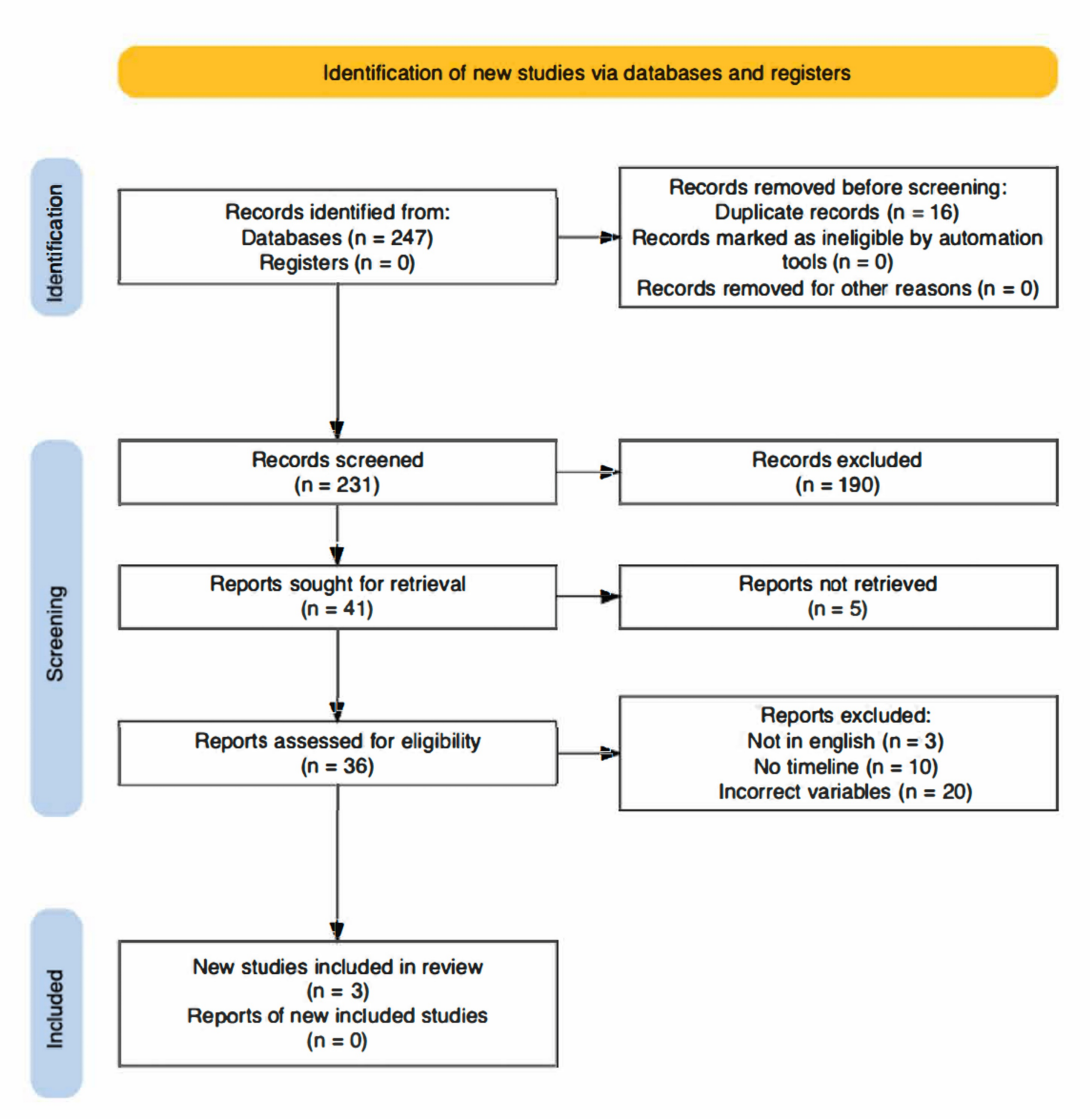
Preferred Reporting Items for Systematic reviews and Meta-Analyses (PRISMA) flowchart.

After a comprehensive search of the literature, 247 articles were initially identified; of these, 16 articles were removed as duplicates. Subsequently, 231 records remained for screening. 190 records were deemed ineligible due to wrong intervention and/or wrong study design. In total, 41 articles were retrieved for full-text review. Of these articles, three were not in the English language, five articles could not be retrieved, 10 articles had no timeline for postoperative results, and 20 articles did not use the variables investigated. Finally, three articles met the inclusion criteria for this meta-analysis (Figure 1).

Results

Three studies (Kang et al. [9], Chan et al. [10], Zhou et al. [11]) met the inclusion criteria for quantitative synthesis. The primary endpoint was operative time (minutes). Effects were calculated as the MD (MAKO - ROSA), such that positive values indicate MAKO procedures took longer. All values are shown in Table 1.

**Table 1:** Study characteristics and study-level effects. M: mean operative time; S: standard deviation; MD: mean difference; SE: standard error; CI: confidence interval; ROSA: Robotic Surgical Assistant

Authors (year)	M1 (ROSA, mean)	M2 (MAKO, mean)	S1 (ROSA SD)	S2 (MAKO SD)	n1 (ROSA)	n2 (MAKO)	MD (minutes)	SE	95% CI (low)	95% CI (high)	Weight (fixed)
Kang et al., 2024 [9]	95.6	90.0	20.0	18.9	95	115	-5.6	2.7	-0.56	-0.01	22.54%
Chan et al., 2025 [10]	102.0	112.0	28.1	31.5	220	752	10.0	2.22	0.17	0.48	73.40%
Zhou et al., 2024 [11]	94.8	112.7	23.0	12.8	20	20	17.9	5.89	0.3	1.62	4.06%

Three studies (Kang et al. [9], Chan et al. [10], Zhou et al. [11]) evaluated the surgical duration of ROSA and MAKO robotic-assisted TKA procedures. The research conducted by Kang et al. showed that MAKO procedures took less time than ROSA procedures by an average of -5.6 minutes (Hedges’ g = -0.29, 95% CI = -0.56 to -0.01). The study by Chan et al. determined that ROSA procedures lasted longer than MAKO procedures by +10.0 minutes (Hedges’ g = 0.32, 95% CI = 0.17 to 0.48). The study by Zhou et al. showed that MAKO procedures required longer surgery duration with an MD of +17.9 minutes (Hedges’ g = 0.94, 95% CI = 0.30 to 1.62).

The fixed-effect meta-analysis showed a small non-significant effect size of Hedges’ g = 0.21 (SE = 0.07, 95% CI = -0.07 to 0.50), indicating that MAKO procedures took slightly longer than ROSA procedures. The random-eﬀect model also shows a non-significant eﬀect size of Hedge's g = 0.26 (SE = 0.34, 95% CI = -1.19 to 1.72). The studies exhibited substantial heterogeneity (Q = 20.07, p < 0.001; I² = 90.0%), which indicated that study-level results did not align consistently. The prediction interval (-1.70 to 2.12) showed the wide range of true effects between different settings.

**Figure 2:**
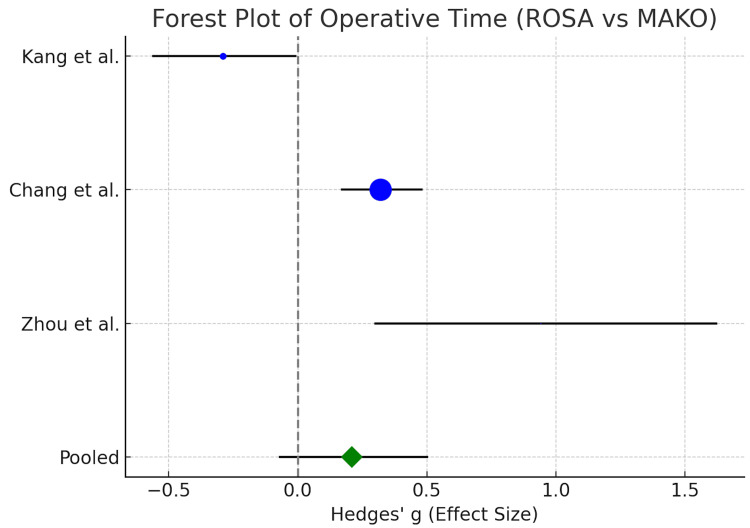
Forest plot of operative times comparing ROSA and MAKO robotic-assisted TKA. Blue circles represent individual studies with 95% CIs, with circle size reflecting study weight. The green diamond shows the pooled effect, where negative values favor MAKO (shorter times) and positive values favor ROSA. Kang et al. [9], Chan et al. [10], Zhou et al. [11]. MD: mean difference; ROSA: Robotic Surgical Assistant; TKA: total knee replacement; CI: confidence interval

The fixed-effect forest plot (Figure 2) demonstrates the contrast between Kang et al., who chose MAKO with shorter times, and Chan et al. and Zhou et al., who selected ROSA with longer MAKO times. The combined data indicate that MAKO procedures require additional time for completion, yet the difference remains statistically insignificant when accounting for heterogeneity.

**Figure 3:**
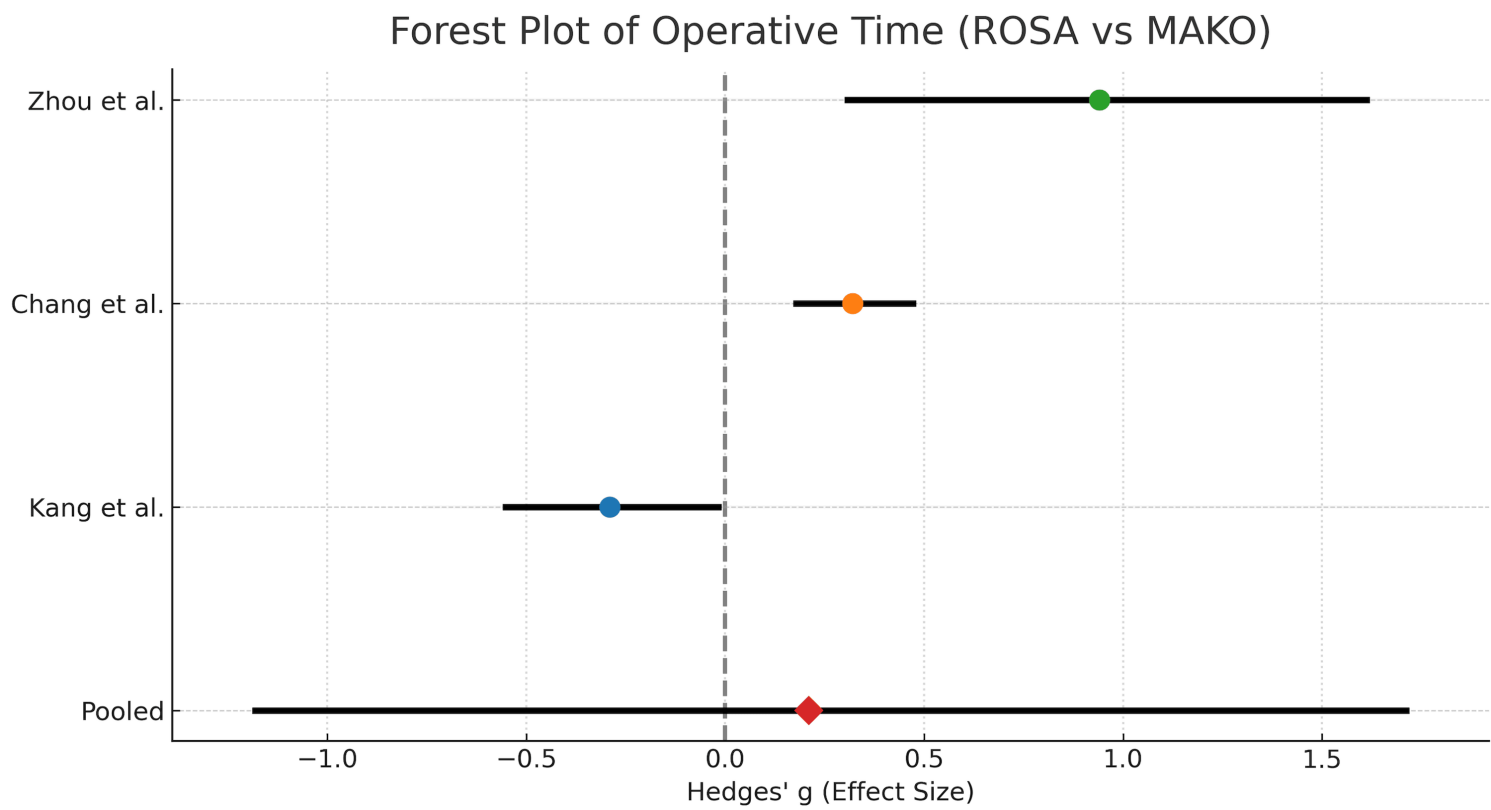
Forest plot displaying the randomized effect sizes (Hedges’ g) and 95% confidence intervals for individual studies, along with their relative weights. The pooled effect size is shown as a green diamond. Kang et al. [9], Chan et al. [10], Zhou et al. [11]. ROSA: Robotic Surgical Assistant

This forest plot (Figure 3) shows the random-effects meta-analysis of mean operative time comparing the ROSA (M1) and MAKO (M2) robotic systems. Individual study effect sizes (Hedges’ g) with 95% CIs are displayed, and the pooled estimate suggests the overall difference in operative time between ROSA and MAKO after accounting for between-study variability. The data for the plot is seen in Table 2.

**Table 2:** Summary of randomized effect sizes (Hedges’ g), 95% CIs, and weights for the included studies. CI: confidence interval

Authors (year)	Hedges’ g	CI lower limit	CI upper limit	Weight (%)
Kang et al., 2024 [9]	-0.29	-0.56	-0.01	36.05
Chang et al., 2025 [10]	0.32	0.17	0.48	38.48
Zhou et al., 2024 [11]	0.94	0.3	1.62	25.47

**Figure 4:**
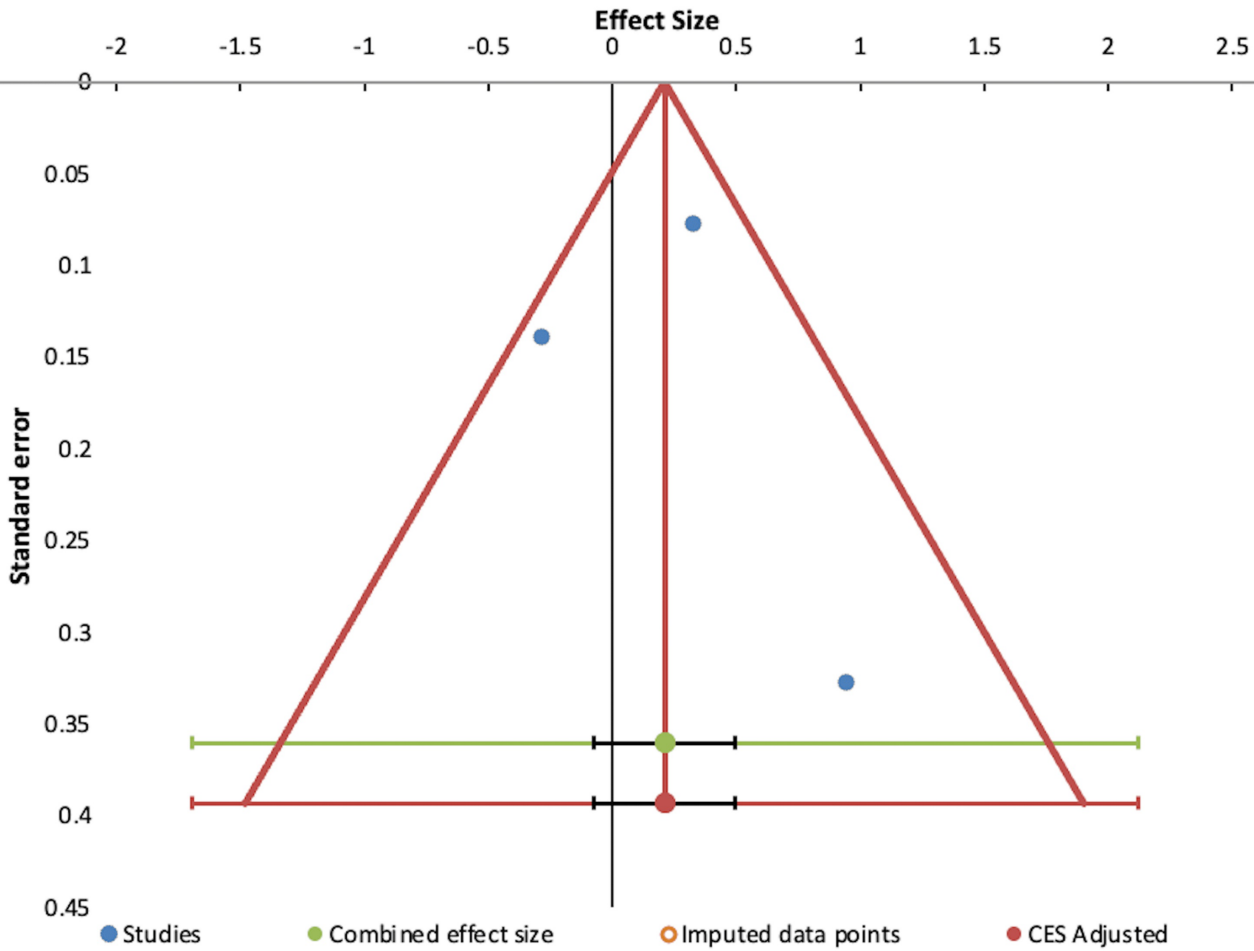
Funnel plot showing publication bias. Blue dots represent included studies, the green dot represents pooled effect size, and red marks the trim-and-fill adjustment. No missing studies were imputed. MD: mean difference

A funnel plot (Figure 4) indicated no missing studies because the studies showed a symmetrical distribution, which suggests minimal publication bias. The observed combined effect size was small (Hedges’ g = 0.21, SE = 0.07, 95% CI = -0.07 to 0.50), and the adjusted effect size remained unchanged after correction, further supporting the stability of the pooled estimate. The studies showed significant heterogeneity (Q = 20.07, p < 0.001; I² = 90.04%), indicating that most of the variability in effect sizes was due to between-study differences rather than sampling error. The wide prediction interval (-1.70 to 2.12) indicates that the expected effects in future studies could be in either a negative or positive direction. The findings are not significantly affected by publication bias, but the high degree of heterogeneity limits the generalizability of the pooled effect size.

Discussion

This review compared operating time intervals between ROSA and MAKO robotic systems for TKA through an analysis of three available studies. The results of the pooled analysis indicated that ROSA procedures had shorter operational durations. The fixed-effects model results demonstrated that MAKO surgical times exceed ROSA times by 6.4 minutes without achieving statistical significance. When using the random-effects model to estimate pooled results, the findings indicated a similar effect direction at +7.3 minutes, yet the CI spanned the line of no difference, resulting in a non-significant outcome.

Each study reported different results regarding the operative time. The results showed that MAKO procedures were shorter according to Kang et al. [9], but Zhou et al. [11] and Chan et al. [10] recorded longer durations for MAKO relative to ROSA. The inconsistent findings between studies produced significant study-level heterogeneity (I² = 68%) because different factors, such as surgeon expertise, institutional procedures, team familiarity, and patient selection criteria, seemed to influence the observed effects.

A difference of 6-7 minutes in operative time appears to have small effects on patient outcomes but may impact hospital resource management when considering large surgical volumes. Operative time represents one factor that affects the overall value of robotic systems in TKA, but other factors, such as accuracy of component placement, learning curves, complication rates, and cost-effectiveness, also play a role.

The learning curve, together with the surgeon’s experience with each robotic platform, represents another essential factor to evaluate. The duration of robotic-assisted TKA surgeries decreases when surgeons and their teams gain experience with the system, which could explain the differences between studies in this meta-analysis. The initial users of robotic technology experience longer surgical durations because they need to perform system calibration and adjust their workflow, but subsequent procedures become more efficient as the technology becomes standard in surgical practice [12]. The reported operative durations in studies show heterogeneity because institutions differ in their resources, and their teams receive different levels of training and follow different perioperative protocols [13]. The contextual factors suggest that variations in operative time between ROSA and MAKO systems stem from both device characteristics and institutional adoption stages, as well as surgeon experience levels. Future research needs to analyze outcomes based on surgeon experience levels and learning phase progression to separate device-specific effects from external factors.

The meta-analysis suggests that ROSA may require less time during TKA procedures than MAKO, although the difference is statistically insignificant, and the available evidence remains inconsistent. More high-quality multicenter randomized or prospective comparative studies need to be conducted to establish the impact of operative time differences on surgical efficiency and patient outcomes.

In addition to operative time, implant alignment and accuracy are critical metrics when evaluating robotic-assisted TKA platforms. According to recent research, the ROSA system achieves precise component placement according to a systematic review, which showed cutting errors below 0.6° in coronal and sagittal planes and better alignment results than traditional methods. Evaluation of robotic-assisted TKA platforms depends on two essential factors, which include operative time and implant alignment [14]. A major research study that analyzed 21 randomized controlled trials through a meta-analysis showed that robotic-assisted TKA achieved better mechanical axis restoration and reduced mechanical alignment errors but required longer surgical times [15]. The main clinical advantage of robotic platforms emerges from their ability to deliver precise and reproducible results, which directly impact implant durability and patient functional results.

Limitations

The analysis faces two major constraints because it relies on a few studies, while showing significant heterogeneity in the data. The restricted number of three trials makes it difficult to evaluate publication bias, and the pooled estimates lack sufficient precision. Study design variations, together with reporting methods, might have led to unmeasurable biases in the data. Overall, more studies are needed to compare the two robotic systems equally. Another limitation of this study is the use of two databases for the initial search.

## Conclusions

This meta-analysis comparing operative times between ROSA and MAKO robotic-assisted systems for TKA suggests that ROSA may be associated with slightly shorter operative times. While the fixed-effects model indicated a statistically insignificant reduction in operative duration with ROSA, the random-effects model, accounting for heterogeneity, showed a non-significant trend in the same direction. The findings highlight variability across studies, underscoring the influence of institutional and surgeon-level factors. Overall, operative time differences between the two platforms appear non-significant and may be of limited clinical consequence in isolation. Larger, well-designed comparative studies are required to confirm these findings and determine whether differences in operative efficiency translate into improved surgical outcomes, cost-effectiveness, or broader clinical value.
